# A Self-Reference Effect on Memory for People: We Are Particularly Good at Retrieving People Named Like Us

**DOI:** 10.3389/fpsyg.2016.01751

**Published:** 2016-11-09

**Authors:** Serge Brédart

**Affiliations:** ^1^Psychology and Neuroscience of Cognition Research Unit, University of LiègeLiège, Belgium

**Keywords:** memory, self, autobiographical memory, personal memory, memory bias

## Abstract

In the present study, it was evaluated whether one’s own name may produce a self-reference bias in memory for people. Results from Experiment 1 indicated that, in a verbal fluency task, participants recalled a greater number of known (familiar or famous) people with the same first name as their own than did paired participants, and vice versa. In the first experiment, paired participants knew each other but were not close. Experiment 2 examined whether this self-reference effect would still occur when the comparison target was a close other. This experiment showed that such a self-reference bias also occurred even when the paired persons were close (partners or very good friends). Overall the present paper describes a new naturalistic case of the self-reference effect.

## Introduction

Over the past 40 years, many studies have suggested that processing one’s own name is prioritized relative to other kinds of social information. One’s own name is a powerful cue for attention: it is more easily perceived as a target and it causes more interference as a distractor (for reviews, see Breska et al., 2011; Humphreys and Sui, 2016). However, it seems that one’s own name gains cognitive priority only when it is presented within the focus of attention or when the participant is set to process it (Gronau et al., 2003; Kawahara and Yamada, 2004; Breska et al., 2011; Yang et al., 2013, but see Alexopoulos et al., 2012).

Recently, Cunningham (2016) suggested that this attentional advantage supports the self-reference effect in memory. Previous studies have repeatedly shown that episodic memory is better for self-related stimuli than for stimuli related to other people, for tasks involving an explicit evaluation of personality adjectives (e.g., “Does the adjective ‘generous’ describe you/the President?”; for a review, see Symons and Johnson, 1997) or for tasks requiring someone to encode objects in a context of self- vs. other-ownership (Cunningham et al., 2008; van den Bos et al., 2010; Turk et al., 2013). One’s own name in itself may produce a self-reference effect on episodic memory: pairings between target stimuli and the self-name have been shown to elicit better memory performance than pairings between a celebrity and target stimuli (Turk et al., 2008). This bias has been found to occur even when the task required participants simply to report whether a word appeared above or below their own name (or a celebrity’s name). Such an incidental effect suggests that we tend to spontaneously form associations between self-related information, such as our own name, and co-occurring external stimuli (see also Sui et al., 2012, Experiment 3D).

In this context, the aim of the present study was to evaluate whether the cognitive advantage for one’s own name may also underpin a self-reference bias in memory for people. We examined whether participants were particularly good at retrieving people with the same first name as their own. It was predicted that, all other things being equal, a participant would retrieve in memory more familiar people with the same first name as their own than a yoked participant would do. For example, imagine that two colleagues David and Simon are paired and they perform a verbal fluency task requiring to recall familiar (famous or personally known) people. David should recall more people called “David” but fewer people called “Simon” than Simon would do.

## Experiment 1

Experiment 1 examined whether, in a verbal fluency task, participants recalled a greater number of known people with the same first name as their own than did paired participants, and vice versa.

### Method

#### Participants

In the absence of previous research on the effect under study, the sample size necessary to evaluate a medium size effect of 0.5 with a power of 0.8 at an alpha level of 0.05 for a two-tailed matched pairs comparison was 34 (G^∗^Power 3.1; Faul et al., 2007). Thirty-four (16 females, 18 males) therefore participated in the study, comprising members of the administrative staff, *post doc* researchers, professors, senior researchers, and students from the University of Liège, aged between 22 and 52 (*M* = 34.0; *SD* = 9.7). The participants’ average educational level, as measured by the number of years of study completed to achieve their highest qualification, was 17.9 (*SD* = 2.5). The sample included 33 French-speaking Belgian and 1 French participants. The mean absolute age difference between pair members was 2.8 (*SD* = 2.6). This study was approved by the Ethics Committee of the Faculty of Psychology, Speech and Language Therapy, and Education of the University of Liège. All participants gave their written informed consent prior to participation.

#### Procedure

To prevent the impact of first name frequency on the self-reference effect, participants were placed in pairs (for example, X and Y) and were asked to recall both people called X and people called Y, so that each name represented a self-related stimulus for one participant and an other-related stimulus for the other participant, and vice versa. Participants within a pair knew each other, were same gender colleagues but were not close to each other (for example they shared no extra-professional activities).

Participants were tested individually and instructed to recall, by writing down on a blank sheet of paper, as many people they knew whose first name was X (or Y) as possible. It was specified that these people could belong to different categories as various as actors, singers, sportspeople, politicians, TV presenters, writers, musicians, characters in novels, cartoons, movies, songs, or famous individuals from any other category, but also non-famous people that they knew personally (these different categories were indicated on a sheet of paper that was placed in front of the participant during the task). Participants were also instructed that there was no obligation to give an exemplar for each category and that giving several exemplars from the same category was allowed. A 5-min time period was allocated for writing down a list of people with each name. For both trials, participants were given advance warning when there was 1 min left to complete the task. Half of the participants first recalled people bearing their own first name and then recalled people bearing the paired participant’s first name, and the other half did it in the reverse order. When a participant recalled a person but was unable to produce that person’s surname he/she was asked to provide precise biographical information about the person, for example “She is my little sister’s best friend” and not simply “She is an acquaintance.” At the end of each trial, the experimenter read each name or description given by the participant and asked the participant to define who each person was (e.g., David Bowie is the singer; Jessica Day is a character in the television series New Girl). This allowed us to disambiguate some responses (e.g., David Copperfield could be either a Charles Dickens’ character or a famous magician) but also to identify people that were unknown to the experimenter.

### Results and Discussion

In the following analysis, the random factor was the participants’ names. In each pair of participants, the number of people named X recalled by participant X was compared with the number of people named X recalled by participant Y, and the number of people named Y recalled by Y was compared with the number of people named Y recalled by X. The participant’s own name and the paired participant’s name were excluded to calculate these numbers (if X’s name was John Smith and Y’s name was Peter Brown, both John Smith and Peter Brown were excluded in calculating the number of names recalled by X or by Y). Only the persons whose first name was phonologically identical to the target name (X or Y) were included, whatever the spelling (e.g., “Katherine,” “Kathryn,” or “Catherine” were all accepted). All analyses were performed using the Statistica 12 software.

Participants reported more people sharing their own first name (*M* = 4.97; *SD* = 2.06) than did their paired participants (*M* = 3.29; *SD* = 1.66), paired *t*(33) = 5.63, *p* < 0.0001, (*M*_diff_ Self vs. Other = 1.68 [95% CI 1.07, 2.28]; Cohen’s *d* = 0.98 [0.48, 1.48]). Over the 169 reported persons sharing the participants’ own name, only one person was a member of a participant’s biological family.

The possibility could not totally be excluded that some participants occasionally cheated by inventing people to enhance their “performance.” To avoid this possible bias, the preceding analysis was rerun on those persons whose existence could be verified, (i.e., the experimenter knew the cited persons or found them on the Internet via Google or on the University Intranet). This analysis also indicated that participants reported more people sharing their own first name (*M* = 3.29; *SD* = 2.05) than did their paired participants (*M* = 2.15; *SD* = 1.35), paired *t*(33) = 4.52, *p* < 0.0001, (*M*_diff_ Self vs. Other = 1.15 [0.63, 1.66]; Cohen’s *d* = 0.79 [0.29, 1.29]).

The first experiment revealed a clear self-reference effect on memory for people: participants could recall more people with the same first name as their own than could paired participants. For example, Simon retrieved more people called Simon than David did, but David retrieved more people called David than Simon did. In this experiment, participants within a pair knew each other but they were not close. Research has shown that the self-reference effect on episodic memory can be diminished or even eliminated when the comparison target is a close other such as a parent, friend, or spouse (Bower and Gilligan, 1979; Symons and Johnson, 1997). In the second experiment, it was evaluated whether or not the self-reference effect on memory for people shown in Experiment 1 would still take place when paired participants were close to each other.

## Experiment 2

Experiment 2 was designed to evaluate whether or not the self-reference effect on memory for people still occurred when paired participants were close to each other.

### Method

#### Participants

Sixteen pairs of romantic partners and one pair of best friends (18 females, 16 males) participated in the second experiment. The mean duration of the relationship was 5.2 years (*SD* = 4.5). These 34 participants were aged between 19 and 54 (*M* = 28.4; *SD* = 8.1), and their average educational level measured by the number of years of study completed to achieve their highest qualification was 16.4 (*SD* = 3.2). The sample included 29 French-speaking Belgians, 3 French and 2 perfect bilingual Luxembourgers. The mean absolute age difference between pair members was 2.9 (*SD* = 3.6). This study was approved by the Ethics Committee of the Faculty of Psychology, Speech and Language Therapy, and Education of the University of Liège. All participants gave their written informed consent prior to participation.

#### Procedure

The procedure was identical to that in Experiment 1 except that participants within a pair were partners or best friends, and they were invited to recall people with the same first name as their own and people with the same first name as their partner/friend.

### Results and Discussion

Participants reported a greater number of people sharing their own first name (*M* = 5.65; *SD* = 2.98) than did their paired participants (*M* = 3.76; *SD* = 1.95), paired *t*(33) = 4.96, *p* < 0.0001, (*M*_diff_ Self vs. Other = 1.88 [1.11, 2.65]; Cohen’s *d* = 0.86 [0.35, 1.37]). Over the 192 reported persons sharing the participants’ own name, only three persons were members of participants’ biological family.

The analysis relating to the persons whose existence was verified also indicated that participants reported more people sharing their own first name (*M* = 3.24; *SD* = 2.09) than did their paired participants (*M* = 1.85; *SD* = 1.28), paired *t*(33) = 4.34, *p* < 0.001, (*M*_diff_ Self vs. Other = 1.38 [0.73, 2.03]; Cohen’s *d* = 0.75 [0.24, 1.24]).

Experiment 2 showed that the effect of self-reference occurred even when pairs of participants were close to each other.

## General Discussion

Previous studies have demonstrated that self-related stimuli, including one’s own name, are particularly powerful cues to attention and produce self-reference effects on episodic memory. The present study showed that one’s own name may induce a self-reference bias in memory for people. Indeed, participants recalled more familiar (famous or personally known) people with the same first name as their own than did paired participants. This difference arose whether paired participants were mere colleagues (Experiment 1) or close persons such as romantic partners or best friends (Experiment 2).

The fact that certain names are more prevalent in certain generations and cultures might have been confounding factors. However, it is quite unlikely that they really were. Indeed, all participants were French-speaking Europeans and the age difference between pair members was on average less than 3 years in both experiments.

This advantage of self-reference over reference to close others is at odds with results of prior studies that tested the classical self-reference effect on episodic memory and reported a reduction or an elimination of the effect when the comparison target was close to the participant (Bower and Gilligan, 1979; Symons and Johnson, 1997). However, consistent with the present study, Sui et al. (2012, Experiment 3D) reported faster responses after self-reference than after reference to close others (best friends) in a task consisting of verifying arbitrary associations between a name (self/best friend) and a geometric shape. It is possible that self-reference is more efficient than reference to close others when tasks do not require an explicit personality evaluation through the activation of a rich elaborative memory representation. In the Sui et al. (2012) task, as in the present study, the self-reference effect may simply result from an attentional advantage at encoding that helped to form associations between one’s own name and co-occurring stimuli [geometrical shapes in the Sui et al. (2012) study or encountered individuals in the present study]. In other words, Cunningham’s (2016) theoretical proposal that the self-reference effect in memory is supported by an attentional advantage at encoding may explain the effect described here. However, the role of retrieval processes in the occurrence of this effect should be tested. This could be done by using divided-attention manipulation at the retrieval phase.

Previous research has reported a self-attention bias for self-related stimuli other than one’s own name, e.g., one’s own face (Brédart et al., 2006; Tacikowski and Nowicka, 2010), hometown, phone number or year of birth (Gray et al., 2004). However, it remains to be evaluated whether one’s own name is particularly prone to elicit a self-reference effect on memory or other self-cues may elicit the effect too. One’s own name possesses several favorable properties. It is a stimulus that people usually like. For instance, people show a preference for the letters occurring in their own names (this preference is known as the Name-Letter Effect; for a review see Hoorens, 2014). It is also an extremely familiar stimulus to which humans are sensitive as early as 4–5 months of age (Mandel et al., 1995; Parise et al., 2010). It has been previously shown that one’s own birthday may induce a self-reference effect: participants were more likely to recall a friend’s birthday when it was close to their own birthday than when it was distant (Kesebir and Oishi, 2010; Rathbone and Moulin, 2010). However, more systematic research is needed to evaluate whether self-related cues other than one’s own name (e.g., year of birth, brand of car) can elicit a self-reference effect on memory for people.

## Conclusion

The results of the present study indicate that we are particularly good at retrieving people named like us.

## Author Contributions

SB: Conceived the design, run the participants, performed the stats, and wrote the paper.

## Conflict of Interest Statement

The authors declare that the research was conducted in the absence of any commercial or financial relationships that could be construed as a potential conflict of interest.
